# Advances in molecular biomarkers for gastric cancer: miRNAs as emerging novel cancer markers

**DOI:** 10.1017/erm.2013.16

**Published:** 2014

**Authors:** Hua-Hsi Wu, Wen-chang Lin, Kuo-Wang Tsai

**Affiliations:** 1 Institute of Clinical Medicine, School of Medicine, National Yang-Ming University, Taipei 112, Taiwan, Republic of China; 2 Department of Obstetrics and Gynecology, Taipei-Veterans General Hospital, Taipei 112, Taiwan, Republic of China; 3 Institute of Biomedical Informatics, National Yang-Ming University, Taipei 112, Taiwan, Republic of China; 4 Institute of Biomedical Sciences, Academia Sinica, Taipei 115, Taiwan, Republic of China; 5 Department of Medical Education and Research, Kaohsiung Veterans General Hospital, Kaohsiung 813, Taiwan, Republic of China

## Abstract

Carcinoma of the stomach is one of the most prevalent cancer types in the world. Although the incidence of gastric cancer is declining, the outcomes of gastric cancer patients remain dismal because of the lack of effective biomarkers to detect early gastric cancer. Modern biomedical research has explored many potential gastric cancer biomarker genes by utilising serum protein antigens, oncogenic genes or gene families through improving molecular biological technologies, such as microarray, RNA-Seq and the like. Recently, the small noncoding microRNAs (miRNAs) have been suggested to be critical regulators in the oncogenesis pathways and to serve as useful clinical biomarkers. This new class of biomarkers is emerging as a novel molecule for cancer diagnosis and prognosis, including gastric cancer. By translational suppression of target genes, miRNAs play a significant role in the gastric cancer cell physiology and tumour progression. There are potential implications of previously discovered gastric cancer molecular biomarkers and their expression modulations by respective miRNAs. Therefore, many miRNAs are found to play oncogenic roles or tumour-suppressing functions in human cancers. With the surprising stability of miRNAs in tissues, serum or other body fluids, miRNAs have emerged as a new type of cancer biomarker with immeasurable clinical potential.

## Introduction

Gastric cancer is one of the most common human cancer types. It is especially prevalent in the Far East region, including Japan, Korea and Taiwan (Refs 1, 2). It is the second leading cause of global cancer death and a malignant disease with high mortality rate in Taiwan (with a mortality rate of 12.7 per 100 000 in male and 9.9 per 100 000 in female populations), despite the declining incidence in the recent decade (Refs 2, 3). In recent studies, surgical resection along with chemo-radiation showed significant improvement over surgery alone (Refs 4, 5). However, most gastric cancer patients have advanced or metastatic diseases at diagnosis (Ref. 6). While surgical resection is an effective therapeutic procedure for curing gastric cancer patients (Ref. 2); the 5-year survival rate is only about 20% for patients with late stage gastric cancer. Although the recent chemotherapeutic regimens have improved the progression-free survival and overall survival of advanced gastric cancer patients, the median survival time is often less than 1 year and none of the regimens has yet emerged as a clear standard (Ref. 7). More research efforts should be emphasised in the early detection of gastric cancers. Therefore, early diagnosis is beneficial and critical for successful surgical removal of gastric cancers since peritoneal dissemination and local/distal metastases often occur in the late stages of gastric cancer and greatly reduce the effectiveness of surgery intervention. Unfortunately, early gastric cancer diagnosis is not feasible for most gastric cancer patients because of the endoscopic gastroscopy that is required to confirm the diagnosis and the lack of useful convenient noninvasive detection biomarkers for routine population screening. In recent years, there have been advancements in the molecular biomarkers utilised in the cancer detection and in the development of therapeutic agents based on the target genes for a few types of solid tumours excluding gastric cancer (Ref. 8). Useful diagnostic biomarkers for early gastric cancer detection remain limited; therefore, it is essential to devote more research to investigate these biomarkers in the near future. This paper discusses the emerging aspect of microRNAs (miRNAs) as a novel gastric cancer biomarker.

As mentioned, it is beneficial to search and identify molecular biomarkers for early gastric cancer diagnosis and disease monitoring. Continuous efforts have been devoted to developing such molecular biomarkers, with over 2000 references related to gastric cancer and biomarker as search keywords listed in the PubMed database during the last decade. Among the reported biomarkers (Table 1), one traditional type is immunological detection based on monoclonal/polyclonal antibodies, which often aims to detect alterations in serological molecules from sera of gastric cancer patients (Refs 9, 10, 11, 12). Carcinoembryonic antigen (CEA) is a frequently used biomarker in the medical examination history of human cancers. The reports of CEA applications in gastric cancers or gastrointestinal tract cancers first appeared in the early 1970s (Ref. 13). Besides CEA, several other protein molecules, CA 19-9, CA 72-4, CA 125 and alpha-fetoprotein, have also been reported to be useful for prognosis and monitoring the recurrence in gastric cancers (Refs 14, 15). However, even with the recent advance of proteomics, almost all of these serum-based biomarkers, including CEA, are not widely recognised in clinical screening or diagnosis because of their limited specificity and sensitivity in early gastric cancer. The exception is pepsinogen testing, which has been used to predict gastric cancer development in East Asia, especially in Japan, for several decades (Ref. 16). The current, commonly used blood biomarkers and their clinical applications are summarised in Table 1.

**Table 1.** Commonly used cancer biomarkers and their clinical applications in gastric cancer.

Biomarkers	Sensitivity rate			Clinical applications		
	Cutoff value	Early stage	Advanced disease	Screening	Prognosis	Monitoring recurrence
Tumour Antigen						
CEA	5.0 ng/ml	<20%	40–50%		●	●
CA 19–9	37.0 U/ml	<20%	20–50%		●	●
CA 72–4	6 U/ml	<20%	30–40%		●	●
AFP	10 ng/ml	*	*		●	●
Hormones						
B-HCG	4 pmol/l	20–35%	30–50%		●	
Protease						
PG I/II	PG < 70 ng/ml; PG I/II ratio <3.0			●		

CEA: carcinoembryonic antigen; AFP: alpha-fetoprotein; B-HCG: free beta-subunit of human choriogonadotropin; PG: pepsinogen (Ref. 10).

*The blood level of AFP increased in hepatoid adenocarcinoma of the stomach or the AFP-producing gastric cancer.

### Molecular biomarkers discovered using molecular biological techniques

With the advancement of molecular biological techniques in the last decades, researchers have gained important insights into the oncogenesis mechanisms. Besides the well-known pathogenic factor, *Helicobacter pylori*, various experimental approaches have identified oncogenes and tumour suppressor genes, including cell cycle regulation genes in the growth and signal transduction pathways (Refs 17, 18, 19, 20, 21, 22). Epigenetic modifications and genome alterations (microsatellite instability) have also been interrogated (Refs 23, 24, 25). Several critical genes have been implicated in different types of gastric cancers (Intestinal type and Diffuse type) and used as potential prognosis biomarkers in previous reviews (Refs 21, 26, 27, 28, 29, 30, 31, 32).

One important type of oncogenes is the protein tyrosine kinases (PTKs) (Ref. 33). Our laboratory has been exploring the utilisation of PTK and protein tyrosine phosphatase genes as biomarkers in human gastric cancers (Refs 34, 35, 36). Various PTKs, the expression of which is elevated in human gastric cancers, have also been studied (Refs 37, 38, 39, 40). We demonstrated that two PTKs, tyrosine kinase with immunoglobulin-like and EGF-like domains 1 (TIE-1) and mitogen-activated protein kinase kinase 4 (MKK4), serve as new molecular biomarkers for gastric cancer prognosis. Expression of TIE-1 kinase in gastric cancer patients is associated with reduced survival rates, and it is an independent factor affecting gastric cancer patient survival (Ref. 37). Moreover, the results revealed that MKK4 is an independent and powerful prognostic factor for gastric cancer progression, especially in the later stages of gastric cancer development (Ref. 38).

**Figure 1.**
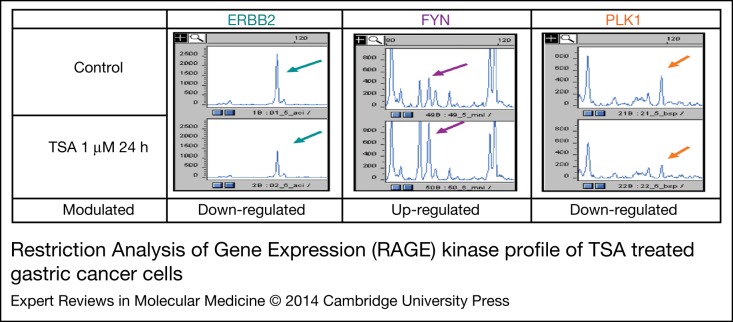
**Restriction Analysis of Gene Expression (RAGE) kinase profile of TSA treated gastric cancer cells.** Human gastric cancer cells (NUGC) were treated with histone deacetylase inhibitor TSA (tritrostatin A) for 24 hours. Total RNA was extracted and used for PTK RAGE analysis. In short, the PTK genes were amplified using degenerate PCR primers designed with PTK conserved motif regions. We labelled the 5′-primer with fluorescent FAM tag. The final PCR product was purified and subsequently digested with various restriction enzymes before conducting the analysis with the capillary electrophoresis sequencer (ABI 3100-avant). ROX labelled size stand and NED labelled house-keeping genes (ALDOA, GPI and LDHA) were mixed and loaded in the electrophoresis chamber. The electrophoresis result was analysed with ABI GeneScan software. Each PTK gene was identified by the respective unique cut restriction enzyme and unique fragment size following capillary electrophoresis. For example, erbB2, fyn and plk1 exhibited significant expression alterations (peaks indicated by the arrows). Among them, fyn is up-regulated and erbb2 and plk1 are down-regulated. This RAGE method allows us to quickly screen the expressed PTK genes with limited tissue samples (only one PCR reaction is needed).

The human genome comprises close to 100 PTKs; hence, it is essential to identify most of the PTKs expressed in cancer cells in order to give a representative picture (Ref. 35). With the advancement of PTK-specific inhibitors (TKIs) as novel therapeutic drug targets, an efficient approach to examine the complexity of PTK expression profiles is beneficial for designing personalised best treatment strategies. Such a general and comprehensive expression profile in a particular cancer type will be also beneficial for molecular diagnosis and prognosis. Since PTK genes are highly conserved from nematodes to humans, sharing significant homologies for their respective kinase catalytic domains, degenerated polymerase chain reaction primers can be designed according to the amino acid sequence submotifs for each kinase catalytic domain (Refs 34, 41). We have adopted this approach by combining special features of the RT-PCR/degenerate-primers for PTK conservative motifs and the differential display technique that separates individual genes by sizing on a sequencing gel. Subsequently, we improved this restriction analysis of gene expression (RAGE) analysis approach with fluorescent-labelled primers and capillary electrophoresis in order to achieve higher throughput and better experimental performance using the auto-sequencing machine. For example, we treated human gastric cancer cell lines with a histone deacetylase inhibitor (TSA) and examined its effect on PTK expression pattern changes. An example of PTK expression detection using this improved RAGE method is shown in Figure 1. Among the modulated PTKs, ERBB2, FYN and PLK1 showed the significant expression level changes in gastric cancer cells treated with TSA. Our data indicated that PLK1 is modulated epigenetically in gastric cancer cells. It is reported that up-regulated PLK1 expression in gastric cancer correlates with a malignant tumour phenotype and has an effect on patient prognosis (Ref. 42).

### Systems biology approaches in the post-genome era

Although the PTK profiling methodology offers an efficient way to screen for possible elevated expression of oncogenic kinases in gastric cancer formation and progression, it is limited to merely a specific type of gene family. With the completion of the human genome project, gene expression profiling has moved into a brand new era. It is now possible to uncover novel molecular tumour markers not elucidated by the traditional and molecular biological methodologies mentioned above. It is feasible to examine several thousand expressed genes and even the entire human transcriptome using SAGE (Ref. 43) and cDNA microarrays (Ref. 44). In the last few years, high-density cDNA microarrays have been widely used to perform genome-wide screening studies of human cancers (Ref. 45). Several studies have also attempted to identify prognostic biomarkers for gastric cancers through genome-wide expression profiling (Refs 46, 47, 48). Thus, molecular staging can be used to define subtypes of gastric cancer based on the gene expression pattern. The molecular classification has been suggested to predict the chemotherapeutic efficacy and relapse in gastric cancers with different gene expression signatures (Ref. 49). Several databases have been established to archive microarray data and interrogate the gene expression information systematically, such as GEO and oncomine, among others. With the power of genome-wide examination of cancer transcriptomes, many novel candidate genes can be identified. Intriguingly, these studies also demonstrated the heterogeneity aspects of human gastric cancers. Often, the candidate gene lists generated from cDNA microarray studies vary considerably among different reports. Therefore, it is essential to interrogate these candidate genes subsequently with other pathological features and validate them systematically by PCR or other independent experimental approaches before utilising them as useful clinical biomarkers.

In addition to the microarray platform, other new exciting technological developments have been introduced to expand our research capacity in terms of genome-wide interrogation and systems biology studies. One example is the proteomic platform established based on the rapid advanced liquid chromatography–mass spectrometry–mass spectrometry (LC–MS–MS). Although still in the early development phase, it already shows great promise for future biomarker discovery. Proteomic profiling has led to the identification of few novel markers for gastric cancer in the last few years (Ref. 50). Novel biomarkers for the prognosis of gastric cancer can also be investigated using serum proteomics with ultra-sensitivity (Ref. 51).

Another critical technology advancement is the MPSS massive parallel sequencing technology. It is often referred to as NGS (next-generation-sequencing), including Solexa (illumina), SOLiD (ABI) and 454 (Roche), among others. NGS provides a new in-depth way to interrogate the genome and transcriptome, and it delivers a sophisticated level of understanding of genome structures and transcriptome variations (alternative splicing). It is astonishing that NGS platform promises even more sequence data with higher throughput and rapid pace in the immediate near future. Whole exome sequencing approach is a popular approach for identifying somatic mutations in cancer genomes. Both Zang et al. (Ref. 52) and Wang et al. (Ref. 53) surveyed the spectrum of somatic mutations of gastric cancer by sequencing the exons of clinical samples and identified frequent mutations in the ARID1A gene locus. In another report, whole kinome sequencing is finished in 14 gastric cancer cell lines by NGS and shows more than 10 604 single-nucleotide-variants (SNVs) in 532 kinase genes, including more than 300 novel kinase SNVs (Ref. 54). Only a limited number of studies focused on the RNA-Seq in gastric cancers (Ref. 55). Kim et al. (Ref. 56) first surveyed whole-transcriptome of 24 samples of gastric tumours and six noncancerous tissues obtained from Asian patients by the NGS approach. Their study revealed that the central metabolic regulator AMP-activated protein kinase (AMPK)α is a potential functional target in gastric cancer in Asians. We have recently applied the NGS platform to the RNA-Seq transcriptome analysis of gastric cancer tissues. We sequenced one pair of gastric cancer tissues by a strand-specific RNA-seq protocol using the illumina HiSeq platform. The normal (adjacent tumour) and tumour tissues comprise 8836563 NGS reads and 89342366 reads, respectively. Following a stringent filter pipeline (with no mismatch allowed, transcriptome-only options) and bowtie–cufflink mapping pipeline (Ref. 57), we obtained a 54 and 41% mapping rate of the NCBI reference gene dataset. Using the cuffdiff analysis package (Ref. 58), we generated a differential expressed gene list with close to 500 genes showing significant expression changes.

**Figure 2.**
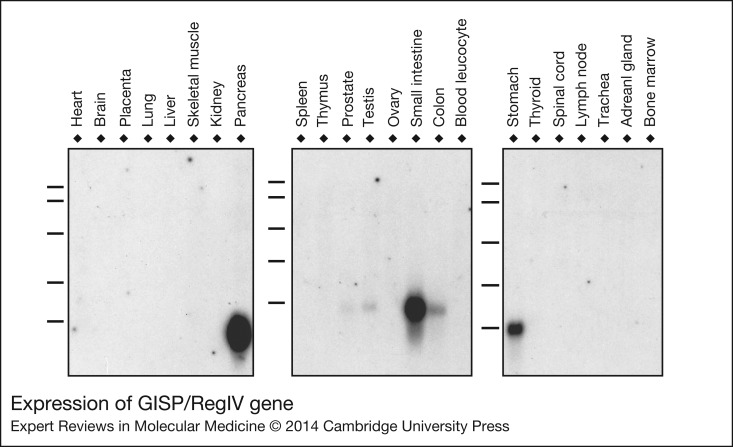
**Expression of GISP/RegIV gene.** Northern blot analysis of GISP/RegIV gene was performed using tissue blots containing total RNA isolated from various human normal tissues. GISP gene was expressed predominantly in the digestive system organs: pancreas, small intestine, stomach, colon, as well as testis tissue.

Gastrointestinal secretory protein (GISP) or REGIV gene, is the top gene candidate that is differentially expressed. We previously identified this gene through bioinformatics interrogation of the human EST database (NCBI accession number AF254415). REGIV is a gastrointestinal tract specific gene expressed mainly in the stomach, small intestine, colon and pancreas by Northern blot analysis (Fig. 2). Recently, REGIV was reported as a bona-fide gastric cancer biomarker in gastric cancer (Ref. 59). REGIV has also been indicated in the resistance of gastric cancer cells to 5-FU treatment (Ref. 60). Therefore, NGS-based RNA-Seq methodology is a powerful tool to interrogate the differentially expressed genes in human gastric cancer samples.

**Figure 3.**
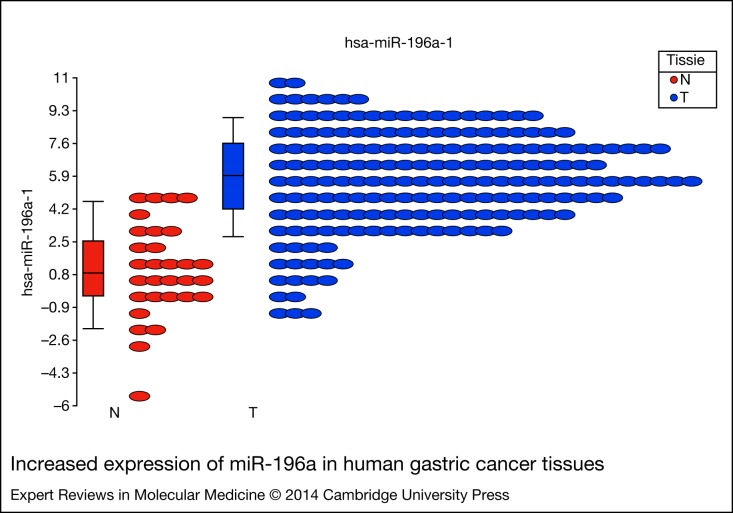
**Increased expression of miR-196a in human gastric cancer tissues.** Human gastric cancer (STAD) miRNA expression NGS short-read level-3 data was obtained from TCGA data portal. In total, we have obtained and analysed miRNA expression profiles of 192 gastric cancer tissues and 22 adjacent-tumour normal gastric tissues using the Partek Genomics Suite (version 6.6). ANOVA statistical analysis was employed and about 33 miRNAs were found to be significantly dysregulated in the gastric cancer (more than 4-fold Fold change and *P* < 0.05). Among them, the miR-196a is significantly overexpressed in the tumour samples.

Furthermore, additional alternative splicing transcript isoforms can be analysed to provide a more comprehensive picture of the cancer cell transcriptome. This new technology will provide more influential studies and in-depth information for future cancer biomarker discovery. An excellent example is the TCGA project (The Cancer Genome Atlas). The Cancer Genome Atlas, initiated in 2006, is taking advantages of the deep sequencing power of NGS in order to promote comprehensive understanding of the molecular basis of cancer through genome, methylome and transcriptome sequencing on several cancer types, including gastric cancer (Ref. 61). One specific aim of the TCGA is to profile the miRNAs expressed in cancer cells (miRNAome). The miRNAs are short and stable single-strand RNA oligomers with newly defined regulatory roles in cells. We have identified a particular miRNA family dysregulated in gastric cancers- miR-196a and miR-196b (Fig. 3), and subsequent experimental analysis demonstrated the epigenetic and transcriptional modulations of miR-196 s in human gastric cancers (Refs 62, 63, 64). Only a few years into their discovery, new and surprising functions and mechanisms have been continuously explored and discovered, especially in tumourigenesis mechanisms and malignant progression (Refs 65, 66). Therefore, much attention has been paid to the miRNAs as emerging cancer biomarkers.

## miRNA: emerging biomarkers for human cancers

### Role of miRNAs in gastric cancer

**Figure 4.**
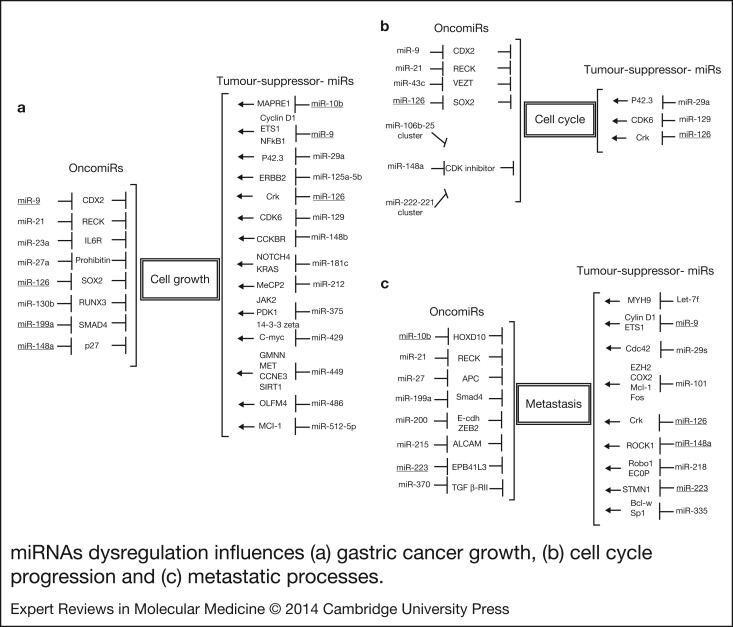
**miRNAs dysregulation influences (a) gastric cancer growth, (b) cell cycle progression and (c) metastatic processes.** OncomiRs promote gastric cancer cell growth and metastasis via inhibition of tumour suppressor genes. Conversely, tumour suppressing miRs suppress gastric cancer cell growth and metastasis by inhibition of oncogenes. The underlined miRNA markers indicate that they play opposite dual functions in gastric cancer.

Consisting of 18–25 nucleotides, miRNAs are a class of nonprotein coding RNA molecules that exert their function by base pairing between the seed region of miRNA and 3′ un-translated regions of (3′-UTR) of target gene. Dysregulated miRNAs play either a tumour-suppressive or an oncogenic role in regulating cell growth, cell cycles and cell migration (Fig. 4a–c), depending on their target genes in gastric cancer. In general, tumour suppressive miRNAs (tumour-suppressor-miRs) usually repress oncogenes and oncogenic miRNAs (oncomiRs) usually silence tumour suppressor genes.

### OncomiRs

The promotion of cancer cell growth is a common feature during gastric cancer progression. Disturbances in the apoptotic pathway result in uncontrolled cell proliferation, which is a critical step in tumour development. OncomiRs are frequently overexpressed in gastric cancer. They promote cancer cell growth and cell cycle progression. They also inhibit apoptosis by silencing growth-inhibition associated genes (Fig. 4). Zhang et al. (Ref. 67) had identified RECK as the direct target of miR-21. Knockdown of miR-21 expression by inhibitor significantly inhibited cell proliferation, migration and invasion as well as increased apoptosis in gastric cancer cells. The expression levels of miR-23a were significantly high in gastric adenocarcinoma tissues, which play a pivotal role in promoting gastric cancer cells growth via silencing IL6R (Ref. 68). High expression levels of miR-130b increase cell viability and reduce cell death by targeting RUNX3 in gastric cancer cells (Ref. 69). The miR-222-221 and miR-106b-25 clusters have been found to be abnormally up-regulated in gastric cancer tissues and reported to suppress the p21 family of CDK inhibitors (p57KIP2, p21CIP1 and p27KIP1), resulting in impaired cell cycle and cell growth (Ref. 70).

Transforming growth factor-β1 (TGF-β1) is known to be involved in the invasion and metastasis in gastric cancer. Overexpression of miR-199a significantly inhibited Smad4, which is a central cellular transducer of TGF-β signalling, significantly inhibited the ability of TGF-β to induce gastric cancer cell growth arrest and apoptosis, and promoted anchorage-independent growth in soft agar (Ref. 71). Down-regulation of prohibition and APC by miR-27a may explain why suppression of miR-27a can inhibit gastric cancer cell growth and metastasis (Ref. 72). Epithelial–mesenchymal transition (EMT) plays a pivotal role in the cancer cell metastasis process. The miR-200 family promotes EMT and can lead to cancer cell migration by impairing E-cadherin and ZEB2 expressions (Refs 73, 74). Moreover, miR-215 inhibits activated leukocyte cell adhesion molecule (ALCAM) expression at the posttranscriptional level and can increase the migration of gastric cancer cells (Ref. 75). Exogenous miR-370 expression can decrease TGFβ-RII expression and the phosphorylation of Smad3 elicited by TGFβ1. Therefore, miR-370 expression can increase gastric cancer migration by disrupting the TGFβ signalling (Ref. 76).

### Tumour suppressive miRNAs

Lost expression of tumour-suppressor-miRs leads to accelerated cell growth, cell cycle progression, and impaired inhibition of oncogenes gene expression (Fig. 4). Cui et al. (Ref. 77) showed that miR-29a could repress p42.3 expression at both the mRNA and protein levels via directly binding to its 3′UTR, resulting in cell proliferation inhibition and cell cycle arrest. The miR-125a has been reported to be a tumour suppressor in malignancies of gastric cancer and to suppress the proliferation of gastric cancer cells in combination with trastuzumab, a monoclonal antibody against ERBB2 (Ref. 78). Hypermethylation silencing miR-129 expression is associated with a poor clinical outcome in gastric cancer while restoration of miR-129 is linked to the cell growth inhibition and stimulation of apoptosis through suppression of CDK6 expression (Refs 79, 80). The expression of miR-181c, miR-212 and miR-512 was silenced with DNA hypermethylation in gastric cancer, and their restored expression could induce decreased gastric cancer cell growth via inhibition of oncogenes expression (Refs 81, 82, 83). Song et al. (Ref. 84) showed the significant down-regulation of miR-148b in gastric cancer tissues, indicating that overexpressed miR-148b could inhibit cell proliferation in vitro and suppress tumourigenicity in vivo by modulating CCKBR expression. Another study revealed low expression levels of miR-375 in gastric cancer tissues, showing that its ectopic expression in gastric carcinoma cells reduced cell viability via suppressing JAK2, PDK1 and 14-3-3zeta, indicating that miR-375 is a candidate tumour suppressing miRNA in gastric carcinoma (Refs 85, 86). Both miR-429 and miR-486 were greatly down-regulated in human gastric carcinoma tissue while cell viability and proliferation were inhibited in miR-429 and miR-486-transfected cells (Refs 87, 88). Restoration of miR-449 in gastric cancer cells led to down-regulation of the GMNN, MET, CCNE3 and SIRT1 genes, which is accompanied by a reduction in cell proliferation (Ref. 89).

Cell invasion and migration were significantly impaired through silencing MYH9 expression in let-7f transfection cells (Ref. 90). Ectopic expression of miR-101 has been shown to significantly inhibit cell growth, cellular migration and invasion of gastric cancer cells via mediating EZH2, COX-2, MCL-1, FOS genes (Ref. 91). Carvalho et al. (Ref. 92) demonstrated that miR-101 gain of function led to a strong depletion of endogenous EZH2 and consequent rescue of E-cadherin membranous localisation. This indicates that miR-101 may function as a tumour suppressor in gastric cancer, as it has an inhibitory role in cellular proliferation and metastasis. Overexpression of miR-218 was implicated in cell growth and metastatic gastric cancer through direct targeting of ECOP and ROBO1 (Ref. 93). Low expression of miR-335 was significantly associated with lymph-node metastasis and invasion of lymphatic vessels. Transfection of pre-miR-335 precursor suppressed gastric cancer cell invasion and metastasis by targeting BCL-w and specificity protein 1 (SP1) but has no significant effects on cell proliferation (Ref. 94).

### miRNAs play opposite dual function in gastric cancer

The function of miRNA depends on its target genes expression. Therefore, a miRNA may play opposite dual functions, either tumour-suppressor-miRs or oncomiRs in gastric cancer. Transfection of a pre-miR-9 precursor significantly silenced CDX2 expression and increased cell growth by facilitating cell cycle progression (Ref. 95). Conversely, miR-9 was frequently down-regulated with DNA hypermethylation in gastric cancer and targeted NF-κB1, cyclin D1 and ETS1 to contribute to the suppression of cancer cell proliferation and metastasis in gastric cancer (Refs 96, 97, 98). In gastric tumour specimens, miR-10b levels were dramatically elevated in lymphoma node metastasis-positive tumour tissues compared to lymphoma node metastasis-free tumour tissues and were found to down-regulate HOXD10 expression (Ref. 99). Kim et al. (Ref. 100) reported that miR-10b was silenced with promoter hypermethylation in gastric cancer cells, resulting in a significant decrease in colony formation and cell growth by repressing MAPRE1 expression.

Previous studies revealed that miR-126 targeting both oncogenes and tumour suppressor genes could play an opposite role in gastric cancer. Feng et al. (Ref. 101) reported that miR-126 potentially inhibited metastasis and cell growth by inducing cell cycle arrest in the G_0_/G_1_ phase through regulating CRK in gastric cancer. Contrasting results revealed that miR-126 directly repressed SOX2 expression by targeting its 3′-UTR and resulted in promotion of cell growth and cell cycle progression (Ref. 102). The same phenomena were observed for miR-148a, which silenced cell cycle inhibitor p27 to result in cell proliferation (Ref. 103). However, other studies revealed that miR-148a was significantly down-regulated in gastrointestinal cancers, and it repressed gastric cancer metastasis by targeting ROCK1 (Ref. 104).

Stathmin1 (STMN1) is a candidate oncoprotein and prognosis marker in gastric cancers. Kang et al. (Ref. 105) reported that miR-223 directly targeted STMN1 expression and repressed cell growth and metastasis. Li et al. (Ref. 106) found that miR-223 up-regulated in gastric cancer, especially in patients with lymph node metastasis at an advanced pathological stage. Moreover, overexpression of miR-223 promotes gastric cancer invasion and metastasis by targeting tumour suppressor EPB41L3 expression. Therefore, numerous miRNAs play a dual function by targeting different genes during gastric cancer progression. Their detailed roles need to be further investigated in the future.

### Clinical implications: miRNAs as diagnosis biomarker

During the past two decades, lack of highly sensitive and noninvasive diagnostic biomarkers led to only modestly improved prognosis for gastric cancer. miRNAs can be released from cancer cells to body fluids via secreting exosomes particles, which could protect them from RNase degradation in circulation. Several circulating miRNAs have been detected in sera, plasma, urine, tears, amniotic fluid and gastric juice (Refs 107, 108, 109). The different expression patterns of circulating miRNA in body fluids might originate from different cell types under certain physiological status (Refs 108, 110). Therefore, miRNA might be a useful noninvasive biomarker for diagnosis and recurrent gastric cancer.

Mitchell et al. (Ref. 109) demonstrated that expression levels of circulating miRNAs in serum are consistent with gastric tumour tissues, and they could serve as a biomarker for cancer detection. In gastric cancer, several circulating miRNAs have been studied as potential diagnostic biomarkers by evaluating their amount in serum, plasma and gastric juice (Table 2). Among them, most studies have focused on individual oncomiRs or tumour-suppressor-miRs, comparing their expression levels in serum or plasma between gastric cancer patients and healthy controls. Several miRNAs circulating in blood of gastric cancer patients can be applied as diagnosis biomarkers, including let-7a, miR-1, miR-17-5p, miR-21, miR-20a, miR-27a, miR-34, miR-106a/b, miR-196a, miR-199a-3p, miR-218, miR-221, miR-223, miR-370, miR-376c, miR-378, miR-421, miR-423-5p, miR-451 and miR-486 (Refs 64, 76, 111, 112, 113, 114, 115, 116, 117).

**Table 2.** Circulating miRNAs as diagnostic biomarkers in gastric cancer.

Body fluid	microRNAs	References
Serum	miR-1, miR-20a, miR-27a, miR-34, miR-196a, miR-378, miR-221, miR376c, miR-423-5p	(64, 114, 115, 116)
Plasma	let-7a, miR-17-5p, miR-21, miR-106a/b, miR-199a-3p, miR-218, miR-223, miR-370, miR-451, miR-486	(76, 111, 112, 113, 117)
Gastric juice	miR-21, miR-106a, miR-129, miR-421	(118, 119, 120)

A few studies have performed systematic miRNAs profiling of blood samples from patients with gastric cancer. Liu et al. (Ref. 114) identified a profile of five serum miRNAs (miR-1, miR-20a, miR-27a, miR-34 and miR-423-5p) as biomarkers for gastric cancer detection, and their expression level correlated well with the tumour stage. In large-scale analysis, the plasma concentrations of miRNAs (miR-17-5p, miR-21, miR-106a and miR-106b) were significantly higher in gastric cancer patients, and they decreased significantly in pre-operative serum compared to post-operative serum (Ref. 117). Song et al. (Ref. 116) identified different miRNAs expression in serum pools of gastric cancer and healthy control using a microarray approach. They identified markedly high levels of 16 miRNAs in gastric cancer compared with controls. Among them, the expression patterns of miR-221, miR-744 and miR-376c in serum could be used as biomarkers to distinguish gastric cancer patients from healthy individual (Ref. 116).

Using microarray profiling on plasma from 20 gastric cancer patients and 20 healthy controls, Li et al. (Ref. 112) found that 37 miRNAs up-regulated and 7 miRNAs down-regulated miRNAs in gastric cancer plasma. Further validation experiments with another 30 gastric cancer patients and 30 healthy controls revealed that miR-199a-3p and miR-151-5p were found to be significantly elevated in gastric cancer patients, and their expression were significantly reduced following the surgery. Konishi et al. (Ref. 111) performed microarray analysis comparing pre- and post-operative plasma of gastric cancer, selecting two candidate miRNAs, miR-451 and miR-486, as plasma biomarkers. In validation, miR-451 and miR-486 significantly decreased in post-operative plasma in 90 and 93% of patients and increased in gastric cancer patients compared with controls.

Chen et al. (Ref. 107) examined the stability of serum miRNAs under harsh conditions, including boiling, low/high pH and freeze-thaw cycles. Surprisingly, serum miRNAs remained stable when treated for 3hr in low (pH = 1) or high (pH = 13) pH solution. Therefore, gastric juice may be used to evaluate expression levels of certain miRNA diagnostic biomarkers that can assist in screening for gastric cancer. Cui et al. (Ref. 118) reported that levels of miR-21 and miR-106a in gastric juice were significantly different between gastric cancer patients and patients with benign gastric ulcers. Zhang et al. (Ref. 119) used real-time PCR to analyse miR-421 expression levels in gastric juice from patients with gastric cancer or benign gastric disease. The results showed that gastric juice levels of miR-421 in patients with gastric cancer differed significantly from the levels observed in patients with benign gastric disease. Yu et al. (Ref. 120) analysed miR-129 expression in 141 gastric juices samples collected by gastroscopy from gastric cancer, gastric ulcer, atrophic gastritis and minimal gastritis patients and subjects with normal mucosa. Their data showed that the miR-129 level in gastric juice was significantly lower in patients with gastric cancer compared with patients with benign gastric diseases. Taken together, circulating miRNAs in blood or gastric juice could be used in the diagnosis of early gastric cancer and might be a remarkable improvement compared to using serum CEA alone.

### miRNAs as prognosis biomarkers

Although the clinical outcome of gastric cancer has been improving gradually, the prognosis of patients in advanced stages and the determination of individuals at high risk in the early stage remain poor. Accumulating recent studies have shown that miRNAs is a promising biomarker that could be used to determine the prognosis of gastric cancer patients and predict the survival rate and recurrence of patients with gastric cancer. Some miRNAs were reported in other cancer types, such as ovarian cancer (Table 3). Metastasis is the major cause of treatment failure in gastric cancer patients. Therefore, it is a challenge to predict the occurrence of metastasis with gastric cancer patients.

**Table 3.** miRNAs as prognostic biomarkers in gastric cancer.

Clinicopathological characteristic	miRNAs	References
Overall survival and recurrence	**OncomiRs:** miR-10b, miR-21*, miR-214*, miR-335*, miR-375	(78, 94, 121, 122, 123, 124, 125, 126, 127, 128)
	**Tumor-suppressor-miRs:** Let-7a*, Let-7 g*, miR-125a*, miR-126, miR-146a, miR-142-5p, miR-223, miR-338, miR-433	

*miRNAs are also reported to be aberrantly expressed in human ovarian cancer.

Increased expression of miR-10b, miR-21 and anmiR-212 was associated with high metastasis risk for gastric cancer patients (Refs 78, 121, 122). Conversely, low levels of miR-125a and miR146a correlated significantly with lymph node metastasis (Refs 78, 123). In general, occurrence of distance metastasis frequently leads to a shorter survival. Therefore, increased levels of miR-10b expression correlated significantly with poor clinical features, including tumour size as well as stage and lymph nodes metastasis. The 5-year survival rate of patients with high levels of miR-10b expression decreased significantly (Refs 121, 124). Elevated miR-21 expression was significantly associated with increased tumour size, lymph node metastasis and decreased overall survival of patients with gastric cancer (Refs 124, 125). Ueda et al. (Ref. 126) reported that low expression of let-7 g and miR-433 and high expression of miR-214 were associated with poor overall survival independent of clinical covariates, including lymph node metastasis and stage. Low expression levels of miR-125a and miR-146a were unfavourable prognostic factor of overall survival (Refs 78, 123). Li et al. (Ref. 124) analysed seven miRNAs expression profiles (miR-10b, miR-21, miR-223, miR-338, miR-30a-5p and miR-126) by real-time PCR in 100 gastric cancer patients and showed that seven-miRNA signatures could predict relapse-free and overall survival of patients with gastric cancer.

Recurrence is a major problem leading to treatment failure and death in gastric cancer patients following surgical resection. Therefore, development of a good biomarker to predict recurrence could greatly improve clinical outcomes. Zhang et al. (Ref. 127) analysed the miRNA expression profile in 65 gastric cancer patients, 29 patients with recurrence and 36 patients without recurrence. Their results indicated that the combination of miR-375 and miR-142-5p could predict recurrence risk for gastric cancer patients. In addition, frequently recurring high levels of miR-335 and poor overall survival correlated significantly with high levels of individual miRNAs in patients with gastric cancer (Refs 94, 128). Although previous studies suggested the use of numerous potential miRNAs as biomarkers in the diagnosis and prognosis of gastric cancer, the values of these miRNAs as biomarkers need to be further confirmed in human gastric cancer patients. The development of a standard protocol for collecting large samples and reanalysing miRNAs in a large independent cohort will be required to validate the clinical significance of selected miRNAs as useful cancer biomarkers.

### Research in progress and conclusion

Gastric cancer is the consequence of a multi-step process resulting from different genetic and epigenetic changes in numerous genes. Dysfunction of oncogenes and tumour-suppressive genes contributes to malignant gastric cancer, and various candidate genes had been implicated to serve as biomarkers for gastric cancer. While biomedical researchers have made many new discoveries leading to numerous publications (evident in the PubMed references), most findings do not translate into useful clinical applications. Translation medicine still requires lots of communication and effort to promote advances in cancer research, especially with high-throughput cDNA microarray and NGS platforms. Even with plenty of putative biomarker genes identified, the outcomes of gastric cancer patients remain dismal due to the modest improvements in clinical treatment strategy. More translational medicine efforts should be made to encourage standardised systematic biomarker validation studies in gastric cancer globally. Otherwise, clinical practices and cancer patients will rarely benefit from the laboratory biomarker discoveries.

With the surprising stability of miRNAs in cancer tissues, formalin fixation and paraffin embedded sections (FFPE), serum, or other body fluids, miRNAs emerge as novel biomarkers for gastric cancer diagnosis and prognosis. The survival and prognosis of gastric cancer patients depends on stage. Unfortunately, it is difficult to detect gastric cancer in the early stage; thus, many patients are diagnosed at advanced stages. Circulating miRNAs in the blood show promise as noninvasive biomarkers in the diagnosis of gastric cancer. It is beneficial to circulate miRNAs and serological protein markers to improve diagnostic sensitivity and increase efficiently monitor recurrence with an aim to improve the survival rate of gastric cancer patients in the future. Distant metastasis is the major problem of treatment failure in cancer patients. Therefore, more sensitive and accurate prognostic biomarkers are essential to determine metastasis and predict prognosis of patients with gastric cancer.

Growing knowledge about the effect of miRNAs on prognostic biomarkers showed that they could accurately estimate metastasis, survival time and recurrences by analysing the miRNA expression level from tissue or FFPE samples. Overall, the analysis of miRNAs expression may provide useful information to assist in decisions of adjuvant therapy after surgical resection. In the future, miRNAs may be promising markers or new therapeutic targets for drug response prediction and control as well as modification of conventional adjuvant treatments. Future clinical studies will need to further confirm the values of miRNA as biomarkers in the diagnosis and prognosis of human gastric cancer.
